# MRI Visibility and MR–DSA Concordance of the Nuvascular Harbor Intrasaccular Occlusion Device: A Preclinical Study

**DOI:** 10.3390/brainsci16040348

**Published:** 2026-03-25

**Authors:** Gökce Hatipoglu Majernik, Andreas Öllerer, Teresa Lassacher, Emre Kaya, Dzmitry Kuzmin, Andrea Janu, Christoph Griessenauer, Monika Killer-Oberpfalzer

**Affiliations:** 1 Department of Neurosurgery, Paracelsus Medical University, Christian Doppler Clinic, 5020 Salzburg, Austria; c.griessenauer@salk.at; 2 Institute of Neurointervention, Paracelsus Medical University, Christian Doppler Clinic, 5020 Salzburg, Austria; a.oellerer@salk.at (A.Ö.); t.lassacher@salk.at (T.L.); m.killer@salk.at (M.K.-O.); 3 Department of Neuroradiology, Paracelsus Medical University, Christian Doppler Clinic, 5020 Salzburg, Austria; e.kaya@salk.at; 4 Department of Minimally Invasive Neurosurgery, Hospital Burgenland, 7400 Oberwart, Austria; dzimitrykuzmin@gmail.com; 5 Department of Neurology, Paracelsus Medical University, Christian Doppler Clinic, 5020 Salzburg, Austria; a.janu@salk.at

**Keywords:** aneurysms, intrasaccular device, MR visibility

## Abstract

**Highlights:**

**What are the main findings?**

- MR-based occlusion assessment demonstrated high concordance with DSA.
- The Harbor intrasaccular device provided consistent and reliable visualization on MR imaging.

**What are the implications of the main findings?**

- MRI may serve as a reliable non-invasive alternative for aneurysm follow-up.
- MR-based follow-up strategies could reduce the need for invasive angiography.

**Abstract:**

**Background/Objectives:** This GLP (Good laboratory practice) study evaluates the MRI compatibility and occlusion performance of the Nuvascular Harbor intrasaccular device for the treatment of bifurcation and sidewall aneurysms in a rabbit aneurysm model. **Methods:** A total of 27 New Zealand White rabbits with 33 surgically created aneurysms (22 bifurcation, 11 side wall) were included and allocated to 90-day (n = 12) or 180-day (n = 15) follow-up. After exclusion of one aneurysm due to parent vessel occlusion and one aneurysm unsuitable for treatment, 31 treated aneurysms remained for analysis. All animals underwent DSA and 3T MRI, including TOF-MRA, FLAIR, DWI, and SWI sequences. Occlusion status was independently graded using the Raymond–Roy Occlusion Classification (RROC), and intermodality agreement was assessed. **Results:** MR-based occlusion assessment demonstrated strong agreement with DSA, with exact Raymond–Roy class concordance in 80.6% of cases and clinically relevant agreement (adequate vs. incomplete occlusion) in 96.8%. Agreement analysis showed substantial concordance (Cohen’s κ = 0.65) and a strong positive correlation (r = 0.79). Adequate occlusion rates were comparable between modalities (87.1% on MRA vs. 83.9% on DSA), supporting the reliability of MR imaging for non-invasive occlusion assessment, reflecting consistent device visibility on MR imaging. **Conclusions:** The Harbor device provides a promising solution for follow up aneurysm occlusion with increased MR visibility, enabling safer, contrast- and radiation-free follow-up. This study emphasizes the need for future endovascular devices to integrate imaging compatibility into their design to enhance long-term patient follow up.

## 1. Introduction

In the era of endovascular therapy, wide-neck aneurysms remain difficult to treat with standard coiling techniques. These lesions are commonly defined by a neck diameter of at least 4 mm or a dome-to-neck ratio below 2 and are frequently associated with unstable coil positioning [1]. Because of their morphology, wide-neck aneurysms often require additional support at the neck, particularly when located at arterial bifurcations, where recurrence after conventional coiling is more common [2,3].

Adjunctive techniques such as balloon-assisted coiling (BAC) and stent-assisted coiling (SAC) were introduced to address these limitations and have improved the feasibility of endovascular treatment in selected cases. However, their use is associated with specific drawbacks. BAC can increase intraprocedural complication risk, while SAC necessitates dual antiplatelet therapy, which might be contraindicated or have a higher risk in the setting of ruptured aneurysms [2]. These limitations have driven the development of alternative approaches, including intrasaccular flow disruption devices designed to treat wide-neck aneurysms without the need for dual antiplatelet medication.

The idea of intrasaccular flow disruption devices first surfaced in the early 2010s with the release of the Woven EndoBridge (WEB) device, which was intended to reduce the need for dual antiplatelet therapy by disrupting intra-aneurysmal flow at the neck and promoting thrombosis without putting material in the parent vessel [4]. The Luna/Artisse, Contour, Medina, Neqstent, Nautilus, Trenza, and SEAL systems are among the several intrasaccular devices that were created after the WEB and obtained CE Mark certification in Europe [5,6,7]. While these devices have significantly expanded the endovascular treatment landscape for intracranial aneurysms, they continue to pose notable challenges, particularly in the context of imaging with magnetic resonance imaging (MRI) and digital subtraction angiography (DSA), which remain the gold standard for follow-up [8].

These limitations prompted the development of the Nuvascular Harbor device, which was purposefully designed with MRI compatibility in mind. At the core of this device is a single-layer Nitinol structure that minimizes the size of the proximal platinum marker at the neck and incorporates mechanical detachment to enhance its visibility on MRI sequences. By incorporating these elements into its design, the Harbor device enables clearer visualization of both the device lumen and surrounding thrombus, potentially improving the reliability of MRI-based follow-up.

To evaluate how the Harbor device performs in a setting that closely resembles clinical use, we employed a preclinical rabbit aneurysm model. This model has been well established in endovascular research due to its anatomical similarities to human cerebral aneurysms [9,10,11,12]. It supports the use of full-scale clinical devices and allows for comprehensive imaging follow-up, including both angiography and MRI. Its value in Good Laboratory Practice (GLP)-compliant studies has been consistently demonstrated, particularly for assessing flow patterns, occlusion behavior, and imaging artifact profiles [9,13].

The Harbor device has been specifically designed to enhance MRI visibility, addressing a key limitation of many currently available intrasaccular devices, which often exhibit susceptibility-related artifacts that may impair reliable non-invasive follow-up. At the same time, its performance must be interpreted within the context of preclinical evaluation, and further studies are required to establish its broader clinical applicability. In this context, we implanted the Harbor device in a GLP-compliant experimental aneurysm model comprising 27 New Zealand White rabbits with 33 surgically created aneurysms.

Our objective was to evaluate whether the device’s MRI-optimized design enables consistent and reliable non-invasive assessment of aneurysm occlusion. Accordingly, we hypothesized that the Harbor device would allow robust and reliable MRI-based assessment of aneurysm occlusion, demonstrating a high level of concordance with DSA. To address this, MR-based occlusion grading was directly compared with DSA findings. Demonstrating high concordance between modalities would suggest that the Harbor device facilitates accurate, radiation- and contrast-free monitoring, thereby addressing a significant unmet need in the long-term follow-up of patients treated with intrasaccular devices.

## 2. Materials and Methods

The GLP24108_NV numbered study was performed in compliance with the GLP regulations at a federally registered test institution with Certificate Reference Number: INS-200015-0005-010. All animal procedures were approved by the competent animal ethics authority (Land Salzburg; approval number: 20901-TGV/128/21-2024) and conducted in accordance with institutional and national animal welfare regulations (animal welfare statement provided at the end of the manuscript).

The study was performed using New Zealand White rabbits and utilized a previously validated microsurgical aneurysm model [14,15,16,17]. To summarize, experimental aneurysms were created using a previously described microsurgical rabbit aneurysm model. Under sterile conditions and operating microscope visualization, the external jugular vein was exposed and harvested as a venous pouch graft. The common carotid arteries were then exposed bilaterally through a midline cervical incision.

For bifurcation aneurysms, the left common carotid artery was ligated proximally, transected, and transposed across the trachea beneath the pretracheal muscles. An end-to-side anastomosis between the left carotid stump and the right common carotid artery was constructed using 10-0 polypropylene sutures (Prolene^®^, Ethicon LLC, San Lorenzo, Puerto Rico 00754, USA; Johnson & Johnson, Norderstedt 22851, Germany), creating a bifurcation configuration. The prepared venous pouch graft was then sutured into the bifurcation apex to form a blind sac aneurysm.

For sidewall aneurysms, a longitudinal arteriotomy was created on the carotid artery, and the venous pouch graft was sutured directly to the arterial wall using 10-0 Prolene. The distal end of the venous pouch was ligated with 7-0 Prolene to form the aneurysm dome. Aneurysm neck width and height were adjusted by the length of the arteriotomy and the position of the ligature. The primary objective was to compare MRI and DSA in assessing the performance of the Nuvascular Harbor occlusion device (HARBOR Occlusion Device, Nuvascular Inc., Irvine, CA, USA).

The experiment was conducted in two consecutive rounds. In the first round, 12 animals were included and randomly assigned in a 1:1 ratio to termination at 90- and 180-days post-implantation. A total of 13 aneurysms were created in this cohort, as one animal underwent implantation of double side wall aneurysms, while the remaining animals received a single aneurysm each.

In the second round, 15 animals were included, with 6 assigned to the 90-day group and 9 to the 180-day group. In the 90-day group, 3 animals received 2 aneurysms; however, in one of these cases, the second aneurysm could not be treated with the device and was therefore excluded from the treated aneurysm cohort. In the 180-day group, 2 animals underwent bilateral aneurysm creation. Consequently, 20 aneurysms were generated in the second experimental series. During control angiography of rabbit 23, complete parent vessel occlusion, likely caused by the surgical creation of the aneurysm, was observed, and this case was excluded from the final occlusion analysis, leaving 18 evaluable aneurysms in this round. Morphologically, the second cohort comprised both bifurcation (n = 9) and side wall (n = 11) aneurysms, allowing assessment of MR and DSA concordance across distinct aneurysm configurations.

**Figure 1:**
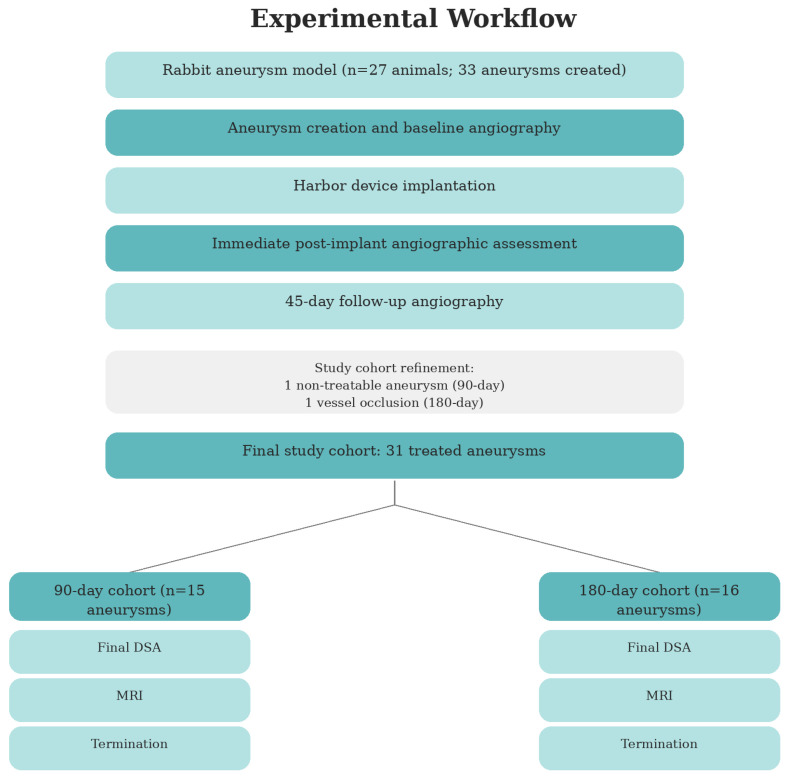
Schematic overview of the study design in a rabbit aneurysm model. A total of 33 aneurysms were created in 27 animals, of which 31 were treated with the Harbor intrasaccular occlusion device. Following aneurysm creation and baseline angiography, device implantation was performed, with immediate post-implant angiographic assessment. A 45-day follow-up angiography was conducted prior to final group allocation. One aneurysm in the 90-day group was excluded as non-treatable, and one vessel occlusion occurred in the 180-day group. The final study cohort comprised 31 treated aneurysms, allocated to 90-day (n = 15) and 180-day (n = 16) follow-up groups. At the respective endpoints, digital subtraction angiography (DSA) was performed, followed by MRI and subsequent termination.

Overall, across both experimental rounds, 27 animals with 33 created aneurysms were included. Of these, 32 aneurysms were treated with the device, and 31 were available for the final occlusion analysis after exclusion of the vessel-occluded case. The experimental flow was demonstrated in Figure 1. All animals were cared for and handled in accordance with institutional animal welfare regulations and in line with our group’s previously published experimental protocols [18].

To ensure precise baseline measurements at the time of embolization, we used an external reference object of known diameter—such as a stainless-steel ball—during imaging. Key anatomical parameters recorded included proximal and distal parent vessel diameters, aneurysm neck width, aneurysm height and width, as well as the dome-to-neck ratio. A single Harbor device was implanted into each bifurcation aneurysm, and for the purpose of assessing vessel occlusion in a non-aneurysmal setting, one additional device was placed in a branch of the external carotid artery. For the double side-wall aneurysm, 2 devices were implanted; one per aneurysm.

Pre-implantation angiographic assessments involved measuring parent vessel diameters (both proximal and distal), along with aneurysm dimensions including aneurysm height and width, neck width, and dome-to-neck ratio. Three-dimensional angiography was performed when requested to better assess complex anatomy.

Device selection was tailored according to aneurysm morphology, and selection was performed by a neurointerventionalist in consensus with a sponsor. All procedures were performed under fluoroscopic guidance using a Siemens Artis Zee monoplane angiography system. Following femoral access, the aneurysm was reached using a 0.027-inch microcatheter (140 cm in length) or 0.017-inch microcatheter (150 cm in length) equipped with a Y-adapter (Tuohy-Borst valve) and connected to a continuous saline flush. Under fluoroscopic guidance, the microcatheter was advanced over a 0.014-inch microwire through the guide catheter in a telescoping technique into the aneurysm lumen. The microwire was subsequently removed. The respective device was then inspected for air bubbles under sterile water, back-flushed, and loaded into the microcatheter. Device deployment into the aneurysm was performed using a combination of push, unsheathing, and loading techniques. Once optimal positioning within the aneurysm was confirmed, the device was detached mechanically. This procedure was repeated for each aneurysm across all animal subjects.

Immediately after implantation, flow disruption and occlusion were evaluated using the Raymond–Roy Occlusion Classification (RROC):

- Class I: Complete occlusion
- Class II: Residual neck
- Class III: Residual aneurysm

Follow-up angiographic imaging was conducted at the respective endpoints (day 90 or 180) using either femoral or ear artery access. DSA images were used to assess aneurysm occlusion based on RROC criteria and to evaluate parent vessel patency.

After completing angiographic assessment, each animal underwent MRI using a Siemens 3T scanner. The purpose was to examine device visibility, determine the degree of aneurysm occlusion, and identify any neurological effects. Animals were imaged under general anesthesia, and a saline-filled bag was positioned beneath the head to improve signal quality. The MR protocol included the following sequences:

- Fluid-Attenuated Inversion Recovery (FLAIR)
- Diffusion-Weighted Imaging (DWI)
- Susceptibility-Weighted Imaging (SWI)
- Time-of-Flight (TOF) MR angiography (MRA)

TOF-MRA was acquired with a repetition time (TR) of 24 ms, echo time (TE) of 4.37 ms, and a flip angle of 18°, using a slice thickness of approximately 0.55 mm and a high-resolution matrix (676), optimized for small-animal vascular imaging. DWI was performed with a TR of approximately 3440 ms and TE of 54 ms using standard diffusion-weighting parameters for ischemia detection. SWI was acquired with a TR of 28 ms and TE of 20 ms, with a slice thickness of approximately 0.7 mm, allowing sensitive detection of susceptibility-related signal changes. FLAIR imaging was included to assess potential parenchymal abnormalities.

All MRI scans were independently reviewed by two neurointerventionalists who were directly involved in the study. They compared the MR findings with the corresponding angiographic data to assess consistency between modalities. In addition, an independent neuroradiologist, who was blinded to group allocation and procedural details, reviewed all MR images and compared them to the angiographic results to provide an unbiased evaluation.

Intermodality agreement between MRA- and DSA-based occlusion grading was evaluated using percentage agreement, Cohen’s kappa coefficient for categorical concordance, and Pearson correlation to assess the ordinal relationship between RROCs. The statistical analyses were performed using R (version 4.5.2).

## 3. Results

In total, 27 New Zealand White rabbits were included, in which 33 aneurysms were surgically created. Of these, 11 were side wall aneurysms, and 22 were bifurcation aneurysms. According to the planned follow-up, 12 animals were assigned to the 90-day group and 15 to the 180-day group. After exclusion of one aneurysm due to complete parent vessel occlusion observed on control angiography and one aneurysm deemed unsuitable for treatment with the study device, the remaining 31 aneurysms were included in the final MR and DSA occlusion analysis.

We compared MR findings to the angiographic images to evaluate both occlusion status and device visibility. FLAIR, DWI, and SWI sequences were evaluated to detect potential ischemic or hemorrhagic complications following device implantation. Across the cohort, DWI did not demonstrate any diffusion-restricted lesions suggestive of acute ischemia. In three cases, minor DWI signal alterations were noted by the blinded neuroradiologist without evidence of diffusion restriction, consistent with susceptibility-related artifacts rather than true pathological findings. Likewise, SWI sequences showed no susceptibility artifacts indicative of microhemorrhage or hemosiderin deposition beyond expected device-related artifacts. FLAIR imaging revealed no pathological parenchymal signal alterations or edema. Overall, multimodal MR imaging showed no evidence of procedure-related ischemic or hemorrhagic complications in the analyzed animals.

Intermodality comparison demonstrated a high level of concordance between MRA and DSA for aneurysm occlusion assessment in this experimental aneurysm model. Using DSA as the reference standard, adequate occlusion (RROC I–II) was achieved in 83.9% of aneurysms compared with 87.1% on MRA. Exact Raymond–Roy class agreement was observed in 80.6% of cases, while clinically relevant concordance (RROC I–II vs. III) reached 96.8%, with only a single discordant case.

Agreement analysis demonstrated substantial agreement (Cohen’s κ = 0.65) and a strong positive correlation (r = 0.79), indicating that discrepancies were largely confined to adjacent occlusion classes rather than clinically meaningful misclassification. These findings highlight the strong agreement between modalities and support the reliability of MR imaging for non-invasive follow-up assessment of aneurysm occlusion in this experimental setting.

Table 1 summarizes the principal MR findings—including DWI, SWI, and FLAIR sequences—alongside the occlusion rates derived from both imaging modalities.

**Figure 2:**
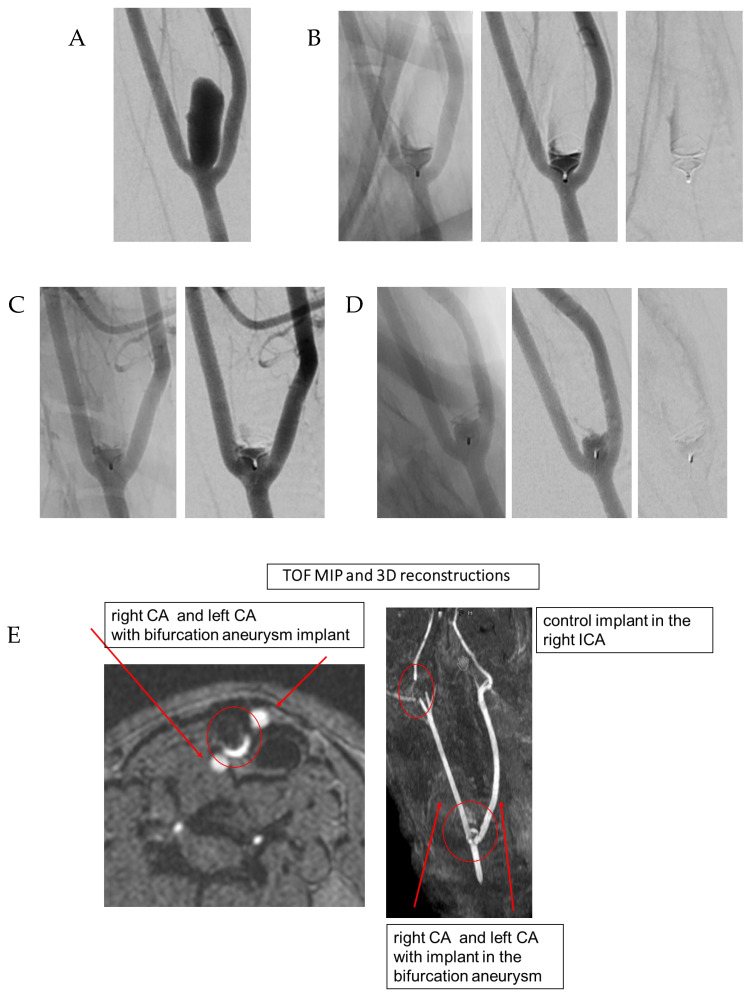

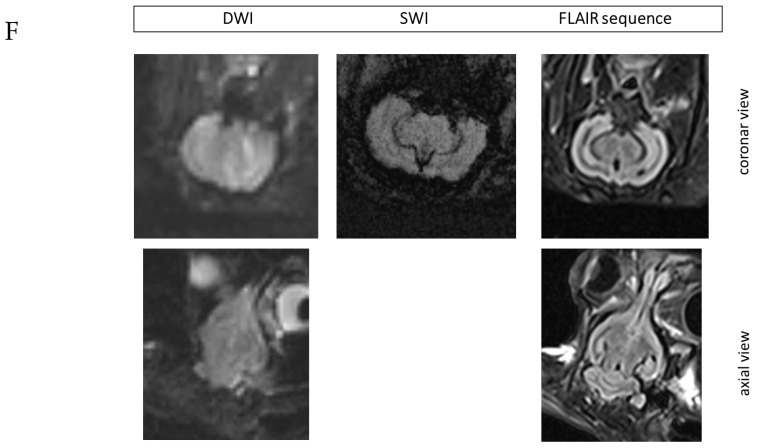
Demonstration of a bifurcation aneurysm in a rabbit 11 (Group 2) with termination at 180 days. Morphological analysis demonstrated a total neck vessel diameter of 7.01 mm, right and left branch vessel diameters of 2.21 mm and 2.54 mm, respectively, a neck width of 3.56 mm, height of 11.82 mm, width of 5.63 mm, and a dome-to-neck ratio of 1.41; the saccular vessel wall measured 15.1 mm and the ECA branch diameter was 1.80 mm. (**A**) Well-established experimental bifurcation aneurysm. (**B**) Immediate post-implantation angiography demonstrating the initial angiographic result. (**C**) Thirty-day follow-up angiogram showing Raymond–Roy class II occlusion. (**D**) One-hundred-eighty-day angiogram demonstrating persistent Raymond–Roy class II occlusion. (**E**) TOF-MRA with MIP and 3D reconstructions correlates with the final angiographic findings. The Harbor device in marked in circle and the carotid arteries were marked with red arrows. (**F**) DWI, SWI, and FLAIR sequences showing no procedure-related complications over the follow-up period.

Figure 2 provides a case example.

## 4. Discussion

### 4.1. Improved MR Visibility with the Harbor Device

**Figure 3:**
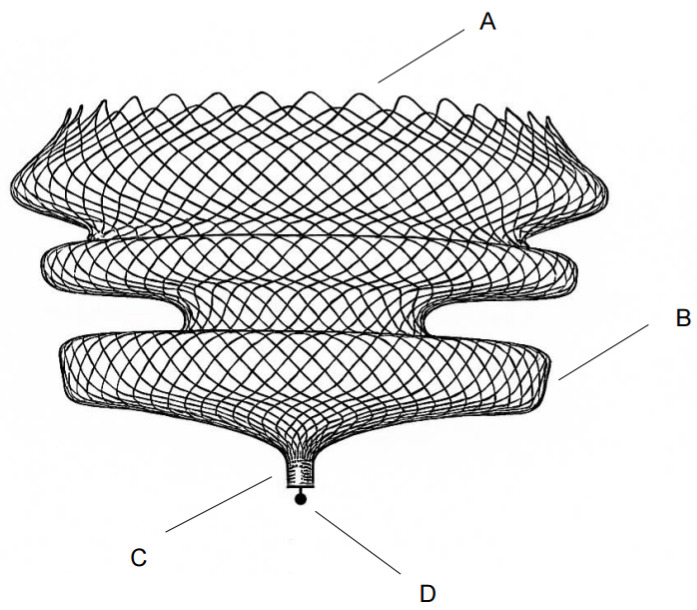
Harbor Intrasaccular Device (A) Open distal end to prevent Faraday cage effect (B) Single layer Nitinol braid to reduce MRI artifact (C) Platinum proximal radiopaque marker 0.5 mm length for enhanced MRI visibility at aneurysm neck (D) Mechanical detachment mechanism, free of ferromagnetic materials.

One of the key observations from this study was the significant improvement in MR visibility provided by the Nuvascular Harbor device (Figure 3). Compared to earlier intrasaccular embolization systems—such as the WEB, Contour, and Artisse—the Harbor device stood out for its consistent signal clarity across MR sequences. This likely comes down to differences in materials, detachment mechanisms, and design.

The WEB device, in particular, has been described as creating a “Faraday cage” effect, which blocks the MR signal almost entirely due to its globular design [19]. The WEB device also has a 1.5 mm long proximal marker band at the aneurysm neck. Although platinum is not ferromagnetic, it is dense and may block or mask the MRI signal, making neck remnants difficult to identify.

The Contour device presents similar limitations. Contour uses an electrolytic detachment mechanism, which incorporates ferromagnetic stainless steel as a sacrificial connector between the implant and delivery system. Remnants of the connector can interfere with the resolution of MRI imaging [20,21]. In addition, its dual-layer Nitinol mesh results in comparable imaging artifacts that obscure both the device’s structure and intra-aneurysmal flow [6,22].

The Artisse device attempts to improve visibility by including platinum marker bands, which help under fluoroscopy. However, its dual-layer nitinol mesh, globular design similar to the WEB, and electrolytic detachment, along with other metallic elements, still lead to substantial susceptibility artifacts on MRI. In most cases, the bands are only faintly visible, while the device and aneurysm lumen remain poorly defined [23,24].

The Harbor device, on the other hand, is a self-expanding, single-layer braided nitinol implant with a cylindrical configuration, incorporating a mechanical detachment system and a short (0.5 mm) proximal platinum marker band. Susceptibility-related artifacts in MRI are driven by local magnetic field distortions at the metal–tissue interface and depend on factors such as metal distribution, structural homogeneity, and device geometry. In this context, the Harbor device showed lower artifact burden and improved lumen definition—particularly on SWI and TOF sequences.

### 4.2. Comparing MR and Angiographic Assessments

Perhaps the most clinically relevant aspect of this study was the comparison between MR imaging and DSA for the evaluation of aneurysm occlusion. Using the RROC, MR-based grading showed a high level of concordance with angiographic findings. This is particularly encouraging in light of the inherent limitations of DSA, including radiation exposure, the need for iodinated contrast agents, procedural invasiveness, and increased time and cost burden. If MRI can provide comparable diagnostic accuracy without these disadvantages, it may substantially influence follow-up strategies after aneurysm treatment.

This consideration is especially important in vulnerable patient populations—such as children, young adults requiring lifelong surveillance, pregnant patients, or individuals with renal impairment—in whom repeated angiographic studies are less desirable. Our findings suggest that future endovascular devices should be designed not only for optimal occlusion performance but also for improved MR compatibility, enabling reliable, non-invasive, long-term monitoring.

Importantly, our results demonstrated a strong concordance between MR- and DSA-based occlusion assessments. Exact RROC agreement was achieved in 80.6% of cases, while clinically meaningful agreement—differentiating adequate from incomplete occlusion—reached 96.8%. Moreover, the rates of adequate occlusion were highly comparable between modalities (87.1% on MRA vs. 83.9% on DSA), underscoring the high predictive reliability of MR imaging for angiographic occlusion status.

These results compare favorably with previously reported data. In a recent study by Nariai et al., evaluating ultra-short echo time MR angiography against conventional DSA in 14 aneurysms, intermodality agreement was 57.1% [25]. In contrast, the markedly higher level of concordance observed in our cohort highlights the reliability of MR imaging of the device for the assessment of aneurysm occlusion in this experimental setting. Taken together, these findings reinforce the potential of MR-based follow-up as a dependable and non-invasive alternative to repeated angiographic examinations, particularly when using devices designed to allow accurate MR visualization.

### 4.3. The Value of the Rabbit Aneurysm Model

Using the well-established rabbit aneurysm model gave us a translationally relevant platform to work with. The anatomy is well-suited to testing devices at a human-like scale, and it allowed us to evaluate how the Harbor device performed under realistic physiological conditions. Across both the 90-day and 180-day end time points, MR visibility remained consistent, suggesting the device maintains its imaging clarity over time. Previous studies have used this model to evaluate devices like the WEB and Artisse, mostly focusing on angiographic outcomes [24,26,27,28,29]. In this study, we focused on not only the angiographic results but also on the correlation with MRI visibility, which, according to our knowledge, has not been systematically addressed in this context before.

### 4.4. A Shift in Device Design Philosophy

A broader implication of this study relates to the evolving philosophy of endovascular device design. Historically, most devices were optimized primarily for performance under fluoroscopy, which explains the widespread incorporation of radiopaque markers. Consequently, MRI has played a limited role in immediate post-procedural assessment. However, as MRI becomes increasingly central in follow-up imaging, particularly with the growing use of advanced techniques such as 4D flow MRI and MR-DSA fusion, there is a clear need for devices that are intrinsically MR-compatible from the earliest stages of design.

The Harbor device exemplifies this paradigm shift by integrating imaging considerations without compromising structural integrity or deliverability. When imaging performance is incorporated into early design decisions, it may be possible to develop devices that function seamlessly across multiple imaging modalities, ultimately benefiting both clinicians and patients.

### 4.5. Limitations of the Study

These findings should be interpreted in light of several limitations. First, although the rabbit aneurysm model offers important experimental advantages, biological differences remain compared with human cerebral vasculature, particularly with respect to flow dynamics and vessel reactivity. Second, while multiple MR sequences were evaluated, more advanced post-processing approaches or AI-assisted reconstruction methods were not explored and might have further enhanced image quality, especially in devices prone to susceptibility artifacts.

In addition, the sample size (n = 27) is relatively small. Although this is common in preclinical GLP studies, a larger cohort might have revealed more subtle differences between MR- and DSA-based assessments. Finally, the study focused exclusively on the Harbor device, and therefore, the generalizability of the findings to other intrasaccular or endovascular devices remains to be determined.

### 4.6. Future Perspectives

The present work lays the groundwork for prospective clinical investigations to more rigorously define the role of MRI as a primary, or at least complementary, imaging modality for follow-up after intrasaccular or endovascular aneurysm treatment. Should MRI-based occlusion grading be standardized across sequences, it may serve as a pragmatic alternative to DSA in selected clinical contexts, particularly where repeated invasive angiography is not desired. Beyond simple occlusion assessment, the integration of advanced MRI approaches, including thrombus characterization and quantitative flow analysis, may allow a more nuanced evaluation of device-aneurysm interaction and the temporal dynamics of aneurysm healing. Such multimodal MRI paradigms have the potential to shift follow-up strategies toward a predominantly non-invasive framework, provided that reproducibility and diagnostic thresholds are validated in larger clinical cohorts.

## 5. Conclusions

From a translational perspective, this preclinical study demonstrates that the Nuvascular Harbor device confers meaningful advantages in terms of MRI visibility and compatibility. The design features of the Harbor appear to mitigate susceptibility-related limitations encountered with conventional intrasaccular constructs, contributing to improved MR visualization in this experimental setting. The observed concordance between MR- and DSA-based assessments supports the notion that MRI could assume a more prominent role in routine post-procedural surveillance, particularly in patients for whom repeated radiation exposure or invasive angiography is less desirable.

Importantly, the use of a well-established experimental aneurysm model enhances the clinical relevance of these findings and supports further clinical evaluation of the Harbor device. More broadly, these data highlight an evolving paradigm in endovascular device development: performance can no longer be judged solely by deliverability and occlusion durability, but must also encompass reliable, high-quality longitudinal imaging. In this regard, devices designed with intrinsic MR compatibility may facilitate more efficient and patient-centered long-term follow-up strategies in contemporary neurovascular practice.

## Figures and Tables

**Table 1:** Comparison of aneurysm occlusion status assessed by magnetic resonance angiography (MRA) and digital subtraction angiography (DSA) in the experimental rabbit cohort (n = 31 treated aneurysms). Occlusion was graded according to the Raymond–Roy Occlusion Classification (RROC). DSA = digital subtraction angiography; MRA = magnetic resonance angiography; DWI = diffusion-weighted imaging; SWI = susceptibility-weighted imaging; FLAIR = fluid-attenuated inversion recovery; no = no pathological finding. In 3 cases the artefact was higher than the other cases.

Rabbit Number	Aneurysm Type	Termination Day	DWI	SWI	FLAIR	Occlusion MRA	Occlusion DSA
1	bifurcation	90	no	no	no	II	III
2	bifurcation	90	no	no	no	II	II
3	bifurcation	90	no	no	no	II	II
4	bifurcation	90	no	no	no	I	II
5	bifurcation	90	no	no	no	I	II
6	double side wall	90	no	no	no	II	II
7	double side wall	90	no	no	no	II	II
8	bifurcation	180	no	no	no	II	II
9	bifurcation	180	no	no	no	II	II
10	bifurcation	180	no	no	no	II	II
11	bifurcation	180	no	no	no	III	III
12	bifurcation	180	no	no	no	II	II
13	bifurcation	180	no	microbleed	no	III	III
14	bifurcation	90	no (Artefact)	no	no	I	II
15	bifurcation	90	no (Artefact)	no	no	I	I
16	bifurcation	90	no	no	no	II	II
17	double side wall	90				not treatable aneurysm	not treatable aneurysm
18	double side wall	90	no (Artefact)	no	no	I	I
19	double side wall	90	no	no	no	II	II
20	double side wall	90	no	no	no	II	II
21	double side wall	90	no	no	no	II	II
22	double side wall	90	no	no	no	III	III
23	bifurcation	180				vessel occluded	vessel occluded
24	bifurcation	180	no	no	no	III	III
25	bifurcation	180	no	no	no	I	II
26	bifurcation	180	no	no	no	II	II
27	double side wall	180	no	microbleed	no	I	I
28	double side wall	180	no	microbleed	no	I	I
29	side wall	180	no	no	no	I	I
30	bifurcation	180	no	no	no	II	II
31	bifurcation	180	no	no	no	II	II
32	double side wall	180	no	no	no	I	II
33	double side wall	180	no	no	no	II	II

## Data Availability

The raw data supporting the conclusions of this article will be made available by the authors on request.

## Abbreviations

The following abbreviations are used in this manuscript:

BAC	Balloon-Assisted Coiling
CE Mark	Conformité Européenne Marking
DSA	Digital Subtraction Angiography
DWI	Diffusion-Weighted Imaging
FLAIR	Fluid-Attenuated Inversion Recovery
GLP	Good Laboratory Practice
MRA	Magnetic Resonance Angiography
MRI	Magnetic Resonance Imaging
RROC	Raymond–Roy Occlusion Classification
SAC	Stent-Assisted Coiling
SWI	Susceptibility-Weighted Imaging
TOF	Time-of-Flight
WEB	Woven EndoBridge
